# A neural network underlying cognitive strategies related to eating, weight and body image concerns

**DOI:** 10.3389/fnhum.2023.1274817

**Published:** 2024-01-22

**Authors:** Cristiana C. Marques, Alexandre Sayal, Joana Crisóstomo, João V. Duarte, Paula Castilho, Kenneth Goss, Ana T. Pereira, Miguel Castelo-Branco

**Affiliations:** ^1^Coimbra Institute for Biomedical Imaging and Translational Research (CIBIT), Institute for Nuclear Sciences Applied to Health (ICNAS), University of Coimbra, Coimbra, Portugal; ^2^Center for Research in Neuropsychology and Cognitive and Behavioral Intervention (CINEICC), Faculty of Psychology and Educational Sciences, University of Coimbra, Coimbra, Portugal; ^3^Institute of Psychological Medicine, Faculty of Medicine, University of Coimbra, Coimbra, Portugal; ^4^Siemens Healthineers, Lisbon, Portugal; ^5^Faculty of Medicine, University of Coimbra, Coimbra, Portugal; ^6^Coventry & Warwickshire Partnership Trust, Coventry Eating Disorder Service, Coventry, United Kingdom

**Keywords:** eating psychopathology, fMRI, self-criticism, rumination, avoidance, self-reassurance

## Abstract

Concerns about food intake, weight and body shape can trigger negatively loaded emotions, which may prompt the use of cognitive strategies to regulate these emotional states. A novel fMRI task was developed to assess the neurobehavioral correlates of cognitive strategies related to eating, weight and body image concerns, such as self-criticism, avoidance, rumination, and self-reassurance. Fourteen healthy females were presented audio sentences referring to these conditions and instructed to repeat these internally while engaging their thoughts with the content of food or body images. Participants were asked to report the elicited emotion and rate their performance. All cognitive strategies recruited a network including the inferior and superior frontal gyri, orbitofrontal and anterior cingulate cortex, insula, and dorsal striatum. These brain regions are involved in emotional, reward and inhibitory control processing. Representational similarity analysis revealed distinct patterns of neural responses for each cognitive strategy. Additionally, self-report measures showed that self-criticism was positively associated with superior frontal gyrus (SFG) activation. Self-compassion scores were negatively correlated with activations in the insula and right putamen, while self-reassurance scores were negatively associated with activity in the orbitofrontal cortex. These findings identify a neural network underlying cognitive strategies related to eating, weight and body image concerns, where neurobehavioral correlation patterns depend on the cognitive strategy.

## 1 Introduction

Humans have evolved with the capacity to store energy in periods of food abundance, preparing for potential periods of scarcity (Rosenbaum and Leibel, 2010). Environmental and societal changes have made calorie-rich foods that are high in fat and sugar widely available. Combined with sedentary lifestyles and stressful life experiences (Swinburn and Egger, 2004), this has contributed to overeating and weight gain, which is now a major societal concern.

Female beauty standards, which emphasize an often unattainable slender and thin body (Engeln-Maddox, 2006), have a powerful influence on women’s self-evaluation (Fairburn, 2008). Sociocultural pressure to conform to these standards may lead to the overvaluation of weight and shape and body image dissatisfaction, resulting in feelings of shame, anger, anxiety or disgust. In an attempt to regulate these difficult emotions, individuals may engage in multiple eating, weight and shape-related behaviors, such as restrictive dieting, excessive exercise, misuse of laxatives, and overeating. They may also rely on several cognitive strategies, such as self-criticism, avoidance and rumination (Duarte et al., 2016; Sharpe et al., 2018). However, these mechanisms maintain disordered eating habits, exacerbate negative affect and are linked to psychopathology (Mellings and Alden, 2000; Nolen-Hoeksema, 2000; Gilbert and Irons, 2005; Verhaeghen et al., 2005; Dunkley and Grilo, 2007; Michael et al., 2007; Surrence et al., 2009; Barnes et al., 2010; Erskine and Georgiou, 2010; Rawal et al., 2010; Goss, 2011; Cowdrey and Park, 2012; Inzlicht and Schmeichel, 2012; Seidel et al., 2016; Lazarus and Shahar, 2018).

Self-criticism refers to a negative and harsh judgment of oneself based on physical appearance, behavior or personality (Gilbert et al., 2004). There have been only a few studies on the neural correlates of self-criticism, and the ones conducted have reported activation in the anterior cingulate cortex (ACC) and lateral prefrontal cortex (PFC) regions (Longe et al., 2010; Doerig et al., 2014), which are associated with executive function, error monitoring and behavioral inhibition (Wittfoth et al., 2009; Nejati et al., 2021). A heightened activation of the insula, amygdala, putamen, caudate nucleus and orbitofrontal cortex (OFC) have also been reported (Longe et al., 2010; Doerig et al., 2014, 2016), indicating difficulties in emotional and reward processing when exposed to self-critical stimuli.

Avoidance entails a refusal to experience unpleasant thoughts and feelings with attempts to avoid them (Hayes, 2004). Individuals who intentionally restrict their caloric intake to lose or maintain their weight tend to suppress thoughts about palatable food (Van Gucht et al., 2014). However, this self-control strategy may backfire and lead to an increase in food consumption (Erskine and Georgiou, 2010). Neuroimaging studies have shown the involvement of the dorsolateral PFC (dlPFC) when trying to suppress such thoughts (Mitchell et al., 2007; Carew et al., 2015; Aso et al., 2016) and the ACC in the detection of conflicts during thought suppression (Mitchell et al., 2007). In a recent study, restrained eaters who suppressed thoughts about food showed reduced activity in the dorsal ACC in association with the choice for more high-calorie foods. This impacts conflict monitoring, suggesting that individuals’ self-control resources may be depleted, leading to food choices incongruent with their restriction goals (Zhang et al., 2021). Furthermore, high-trait chocolate cravers exhibited higher activation in the putamen and ventral striatum than those with a low-trait when exposed to chocolate images, indicating the involvement of reward-related striatal areas in craving. Interestingly, high-trait chocolate cravers reported a decreased in chocolate-related thoughts after receiving suppression instructions; however there was no differential neural activity between high-trait and low-trait chocolate cravers (Miedl et al., 2018). The OFC is also implicated in the devaluation of chocolate when frequent chocolate eaters have to avoid it (Lender et al., 2023).

Rumination is a repetitive cognitive process that involves focusing on the causes, symptoms and consequences of distress (Nolen-Hoeksema, 1991). Individuals who engage in ruminative thinking about their eating, weight and body shape experience negative emotions, which can maintain eating psychopathology (Rawal et al., 2010; Cowdrey and Park, 2012; Seidel et al., 2016). An fMRI study that induced rumination by asking participants to recall negative autobiographical memories showed activation in the subgenual ACC and medial PFC (Kross et al., 2009). Cooney et al. (2010) also found that rumination recruited limbic and prefrontal regions, such as OFC, ACC, dlPFC, and amygdala. Additionally, body and weight-related rumination was associated with activation in the ventral striatum, observed in patients with anorexia nervosa (Seidel et al., 2018; Erdman et al., 2020).

As opposed to these defensive cognitive processes, research has found the benefits of self-reassurance and self-compassion. These are linked with the ability to be more understanding, warm, encouraging and supportive toward oneself when facing setbacks (Gilbert et al., 2004, 2017). When individuals are exposed to signals and stimuli such as touching, stroking, holding, soft voice tone, and facial expressions, this activates the soothing affect regulation system and the parasympathetic system, inducing feelings of safeness and contentment (Gilbert, 2014; Carter et al., 2017; Kim et al., 2020b). Research on this topic has highlighted the link between self-reassurance and self-compassion with wellbeing and interpersonal functioning (MacBeth and Gumley, 2012; Yarnell and Neff, 2013) and a reduction in psychopathology (Raes, 2010; MacBeth and Gumley, 2012; Krieger et al., 2013; Petrocchi et al., 2019). Self-compassion can help address negative body image, shame, self-criticism and self-disgust in individuals with eating difficulties (Adams and Leary, 2007; Breines et al., 2014; Ferreira et al., 2014; Kelly and Carter, 2015; Braun et al., 2016; Serpell et al., 2020; Marques et al., 2021). It can also contribute to weight management and appropriate nutrition through healthier eating behaviors (Albertson et al., 2015; Sirois et al., 2015; Rahimi-Ardabili et al., 2018). A recent meta-analysis of compassion with sixteen fMRI studies identified several brain regions related to compassion—middle frontal gyrus, bilateral inferior frontal gyrus (IFG), bilateral insula, ACC, medial frontal gyrus, and basal ganglia/thalamus—which are involved in reward and emotional processing (Kim et al., 2020a).

Taken together, current evidence of the brain regions implicated in self-criticism, avoidance, rumination, and self-reassurance or self-compassion suggests a possible overlap, with consistent activations observed in dorsal and ventral PFC, ACC, insula, dorsal striatum (caudate and putamen), and OFC. However, there is a paucity of studies specifically investigating these cognitive strategies related to eating, weight and body image concerns. To address this gap, in the present study, a new fMRI paradigm was designed to investigate the neural correlates of self-criticism, avoidance, rumination and self-reassurance related to eating psychopathology. During the experimental task, participants were presented with audio sentences related to each cognitive process with eating, weight, and body-related content, while viewing food and body images.

We aimed to investigate whether there were differences in neural activity involved in self-criticism, avoidance, rumination and self-reassurance scripts and their relation to behavioral profiles. We also sought to explore whether such strategies lead to differential patterns of brain activation. Based on previous literature, we expected that self-criticism, avoidance, rumination, and self-reassurance would be associated with the recruitment of superior and inferior prefrontal areas, OFC, dorsal striatum and regions of the salience network, e.g., the insula and ACC. Specifically for the self-reassurance and self-criticism contrast, we expected to observe activation changes in lateral PFC, ACC, caudate, and insula, similar to previous studies (Longe et al., 2010; Kim et al., 2020b). In sum, we aim to identify the neural circuitry underlying distinct but related cognitive strategies featuring self-criticism, avoidance, rumination and self-reassurance regarding eating, weight and body image concerns.

## 2 Materials and methods

### 2.1 Participants

Fourteen healthy females, with a mean age of 24.07 (*SD* = 3.17; ranging from 19 to 29 years) were recruited to take part in the study. The mean body mass index (BMI) was 22.44 (*SD* = 2.74), ranging from 19.05 to 28.08. The inclusion criteria were being female and above 18 years of age. All participants were right-handed, as confirmed by the Edinburgh Handedness Inventory (Oldfield, 1971). The exclusion criteria were having a history of a psychiatric disorder, including eating disorders, or a history of drug or alcohol abuse, taking psychiatric medication, and contraindications to MRI. Participants were recruited through advertisements and word-of-mouth. All volunteers gave their written informed consent to participate in this study. The study was conducted in accordance with the Declaration of Helsinki and was approved by the Ethics Committee of the Faculty of Psychology and Educational Sciences of the University of Coimbra.

### 2.2 fMRI paradigm

**FIGURE 1:**
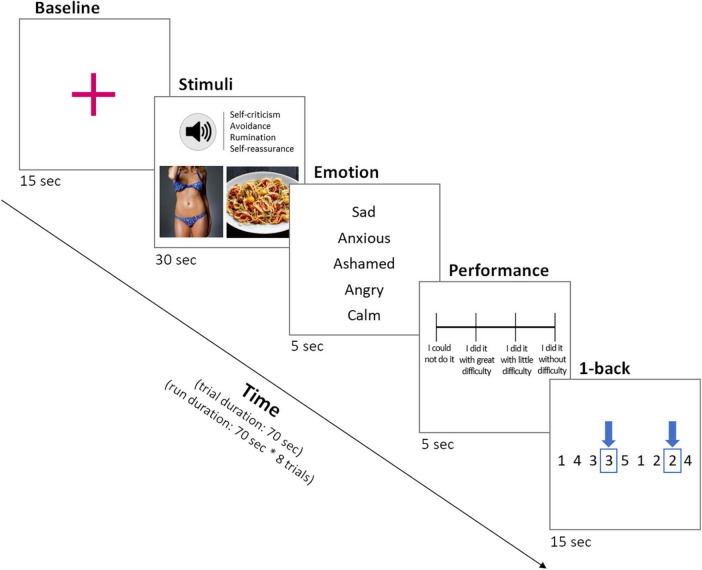
Schematic representation of the fMRI task during a trial.

A block design fMRI experimental paradigm was used to examine participants’ neural responses to different cognitive strategies related to eating and body concerns (see Figure 1). Each trial started with a 15-s presentation of a fixation cross (baseline) to prepare the participant for the upcoming task, followed by a 30-s image presentation of a body or food. Those images were retrieved from public internet sources and the head was removed from each body image. An audio sentence was played once for the first 4 s of the image presentation, which was tailored to one of four cognitive strategies: self-criticism, avoidance, rumination, and self-reassurance. Each strategy was associated with specific content and tone of voice: self-criticism (“I don’t like my body!” in a harsh and hostile voice tone), avoidance (“I’ll think of good things” in an anxious and compelling voice tone), rumination (“Why I have this body?” in a worried voice tone), and self-reassurance (“It’s not my fault for having the body I have” in a warm and caring voice tone). Audio files were recorded by a professional actress, who had training in clinical psychology. For the remaining 26 s, participants were instructed to repeat the sentence internally, using the same tone of voice as the audio, and engage with the cognitive process.

After the image presentation, participants were asked to identify, via a button press (within 5 s), the predominant emotion they experienced during the previous block, among five emotions displayed (sad, anxious, ashamed, angry, calm). Following the emotion rating, participants rated their performance in following the instruction to engage in the cognitive strategy, via a button press (within 5 s). The rating scale had four levels: “I did it without difficulty,” “I did it with little difficulty,” “I did it with great difficulty,” and “I could not do it.” Finally, participants performed a 1-back distractor control task for 15 s. During this task, a series of numbers were displayed on the screen one-by-one, and participants pressed a button whenever the current number matched the immediately previous one. The 1-back task required working memory and attentional processes, which helped participants washout the previous strategy and shift the attentional focus from the prior cognitive strategy engagement task to the present one.

There were 5 task runs, lasting 9.5 min each. Each run contained 8 experimental trials that lasted 70 s each. For each cognitive strategy, 10 trials were presented. In order to control for the confounding effect of images, the same image was displayed four times, one for each condition. For each run, the sequence of conditions and images was randomized to prevent habituation.

Participants were presented with images of either food or body for each condition, as we assumed that under this experimental context both types of images would evoke similar reward and affective brain activity processes. Our primary focus was on exploring the affective processes when participants engaged in eating and body-related self-criticism, avoidance, rumination, and self-reassurance.

### 2.3 Psychological measures

The Self-Compassion Scale (SCS; Neff, 2003) is a 26-item questionnaire that assesses self-compassion. SCS is a five-point Likert scale and the responses vary from 1 (almost never) to 5 (almost always). The internal consistency obtained for the total scale in this study was excellent (α = 0.96).

The Forms of Self-criticizing/Attacking and Self-reassuring Scale (FSCRS; Gilbert et al., 2004) is a 22-item measure to assess self-criticism and the ability to self-reassure when facing setbacks and failure. The responses are given on a five-point Likert scale, ranging from 0 (not at all like me) to 4 (extremely like me). The FSCRS is composed of three subscales, namely, inadequate self, hated self, and reassured self. In this study, hated self was not used, given the low manifestation in healthy individuals. In this sample, the internal consistencies were good for inadequate self (α = 0.91) and reassured self (α = 0.87).

The Ruminative Response Scale for Eating Disorders (RRS-ED; Cowdrey and Park, 2011), a nine-item measure, was used to assess rumination relating to food, weight and body shape. Responses ranged from 1 (almost never) to 4 (almost always). A good Cronbach’s alpha of 0.83 was obtained for the total scale in this study.

The Food Thought Suppression Inventory (FTSI; Barnes et al., 2010) is a 15-item measure that assesses food-related thought suppression. The FTSI is scored on a five-point Likert scale, ranging from 1 (strongly disagree) to 5 (strongly agree). An excellent Cronbach’s alpha of 0.96 was obtained in this study for the total scale.

The Eating Disorder Evaluation Questionnaire (EDE-Q; Fairburn and Beglin, 1994) is a 28-item instrument to assess eating disordered symptoms. The 22 items used to calculate the global score of EDE-Q are answered using a 7-point Likert scale, from 0 (no days/none of the times) to 6 (every day/every time). The Cronbach’s alpha obtained in the present sample was 0.90 for the global scale.

### 2.4 MRI data acquisition

Brain images were acquired at the Institute of Nuclear Sciences Applied to Health (ICNAS) using a 3T Siemens Prisma^fit^ scanner with a 64-channel receive head coil. High-resolution T1-weighted images (repetition time = 2500 ms, echo time = 2.15 ms, flip angle = 8°, field of view = 256 mm, 192 slices, voxel size = 1 mm × 1 mm × 1 mm), were obtained for each participant. Functional data were acquired using a T2* weighted multiband Echo Planar Imaging (EPI) pulse sequence with an acceleration factor of 6 (repetition time = 1000 ms, echo time = 37 ms, flip angle = 52°, field of view = 200 mm, 72 slices, slice thickness = 2 mm, voxel size = 2 mm × 2 mm × 2 mm). For each run, a total of 570 functional volumes were obtained. The first 10 volumes of each run were discarded. We also acquired spin echo field maps for each participant, to minimize distortions due to variations in the magnetic field, with the following imaging parameters: TR = 8000 ms, TE = 66 ms, flip angle = 90 degrees, FoV = 200 mm, 72 slices with slice thickness of 2 mm.

### 2.5 MRI data pre-processing

Results included in this manuscript come from preprocessing performed using *fMRIPrep* 20.2.1 (Esteban et al., 2018a; RRID:SCR_016216; Esteban et al., 2018b), which is based on *Nipype* 1.5.1 (Gorgolewski et al., 2011, 2018; RRID:SCR_002502). For step-by-step detailed information see Supplementary material.

The nifti output of *fmriPrep* was converted to BrainVoyager-compatible formats using a custom script using MATLAB and NeuroElf v1.4.^1^ In BrainVoyager, each functional dataset was high-pass filtered using a cut-off frequency of 0.007 Hz and spatially smoothed with a gaussian kernel of FWHM = 4 mm.

### 2.6 Statistical data analysis

Statistical fMRI data analyses were performed using BrainVoyager 21.4.5 (Brain Innovation, Maastricht, Netherlands; Goebel et al., 2006). Individual subject data were first analyzed separately using general linear models (GLM). The mean effects obtained for each subject were then combined at the group level in a random effect analysis, which accounts for inter-subject variability. One-sample *t*-tests were applied to investigate group effects. Whole-brain analyses were performed for the balanced contrast 1: self-criticism + avoidance + rumination + self-reassurance > baseline, and contrast 2: self-criticism + avoidance + rumination + self-reassurance > 1-back task. The statistical map was obtained using an FDR adjusted, *q* = 0.01 at the single voxel level, and *p* = 0.05 corrected for multiple comparisons at the cluster level. The cluster extent was estimated based on Monte Carlo simulations (1000 iterations), and the minimum cluster size was 26 contiguous voxels for contrast 1 and 27 for contrast 2. The whole-brain analysis of contrast 1 was used to functionally identify region of interest (ROI) from which we extracted the BOLD signal change for each condition. Mean beta weights and *t-*values were extracted for each condition within each ROI, by contrasting each condition relative to the baseline. A two-way repeated-measures ANOVA was run to determine the interaction effect between different ROI and different conditions on brain activity, and its main effects. All coordinates are reported in MNI space. Brain regions were labeled according to the automated anatomical labeling atlas, third version (AAL3) (Rolls et al., 2020b) using MRIcron.

A representational similarity analysis (RSA) was computed using Pearson correlational distance to calculate the degree to which voxel patterns are similar across conditions within each ROI (Kriegeskorte, 2008). A representational dissimilarity matrix (RDM) was calculated for each ROI using the condition responses estimated in the GLM analysis. Therefore, trials of the same condition were grouped in the general linear model. To determine the statistical significance of each measure of dissimilarity between conditions, the following rationale was used: if two conditions would yield perfectly correlated spatial patterns of response (*r* = 1), the corresponding dissimilarity measure would be 0 (distance = 1–*r*). Thus, to examine whether the response pattern for each pair of conditions is statistically significantly different from 0, we tested the dissimilarity across subjects using the individual-level RDMs against 0. Data presented a normal distribution and one sample *t*-tests were employed. Bonferroni’s-adjusted *p*-values were calculated to correct for multiple comparisons (uncorrected *p*/48).

The chi-squared test was calculated to compare the proportions of emotional and performance ratings between images of food and bodies. Correlation analyses between self-reported psychological measures and the beta values for brain activity in the regions of interest were calculated using the IBM SPSS Statistics for Windows, version 27 (IBM Corp., Armonk, NY, USA). Pearson correlation coefficient was used for normally distributed data and Spearman’s rank correlation coefficient when the data were non-normally distributed. The ROIs were selected based on our study hypotheses, which comprise superior and inferior frontal areas, OFC, insula, dorsal striatum and ACC.

## 3 Results

### 3.1 Self-reported psychological measures

Participants showed low to moderate levels of self-criticism, ranging from 1 to 29 (*M* = 13.57; *SD* = 9.00). Scores on the food thought suppression ranged from 15 to 57 (*M* = 23.07; *SD* = 12.37), indicating that participants presented low to medium scores. Scores on the rumination relating to food, weight and body shape ranged from 9 to 22 (*M* = 12.21; *SD* = 4.06), which fell within the normal range. Participants’ eating psychopathology scores were low to medium, ranging from 1 to 55 (*M* = 17.43; *SD* = 16.23). Moreover, they exhibited moderate levels of reassured self, ranging from 12 to 31 (*M* = 23.71; *SD* = 5.36) and self-compassion, ranging from 51 to 115 (*M* = 83.79; *SD* = 19.34).

### 3.2 Behavioral data

#### 3.2.1 Performance ratings

For each trial, participants were asked to rate their performance in the task (engagement with the cognitive process). For the majority of trials in all conditions, participants reported no difficulty in performing the task, ranging from 72.14% of trials for self-reassurance to 81.43% of trials for self-criticism. There were no significant differences in the performance ratings between images of food and body (see Table 1).

**TABLE 1:** Frequencies of emotion and performance ratings for each condition and image type.

	Self-criticism %	Avoidance %	Rumination %	Self-reassurance %	Body %	Food %	χ ^2^	*p*
**Emotion**								
Sad	20.71	9.29	13.57	8.57	17.15	8.93	7.62	0.006
Anxious	10.71	9.29	17.14	1.43	8.93	10.36	0.18	0.668
Ashamed	3.57	11.43	8.57	2.14	7.86	5.00	1.45	0.228
Angry	30.71	12.86	20.00	6.43	12.50	22.50	9.02	0.003
Calm	34.29	57.14	40.71	81.43	53.57	53.21	0.00	1.00
**Performance**								
I did it without difficulty	81.43	75.00	79.29	72.14	77.50	76.43	0.04	0.841
I did it with little difficulty	9.29	12.86	16.43	12.14	13.57	11.79	0.26	0.611
I did it with great difficulty	7.86	7.14	3.57	2.86	4.65	6.07	0.32	0.573
I could not do it	1.43	5.00	0.71	12.86	4.29	5.71	0.34	0.561

#### 3.2.2 Emotional ratings

After the engagement with the cognitive process while looking at an image of food or body, participants were asked to choose the emotion elicited with this task. For all four conditions, the emotion most reported was calmness, however, as expected, the percentages differ between conditions (34.29% of trials for self-criticism, 57.14% of trials for avoidance, 40.71% for rumination, and 81.43% for self-reassurance). Emotion ratings were in general similar with two notable exceptions. There were indeed significant differences in emotional ratings only regarding feelings of sadness for body images and angriness for food, with a significantly higher proportion of participants reporting feelings of sadness for body images, while a higher proportion of participants reported feeling angry for food images (see Table 1).

### 3.3 fMRI data

#### 3.3.1 Contrast 1: self-criticism + avoidance + rumination + self-reassurance > baseline

All regions with significant signal changes with the exploratory whole-brain RFX-GLM group analysis are reported in Supplementary Table 1.

**FIGURE 2:**
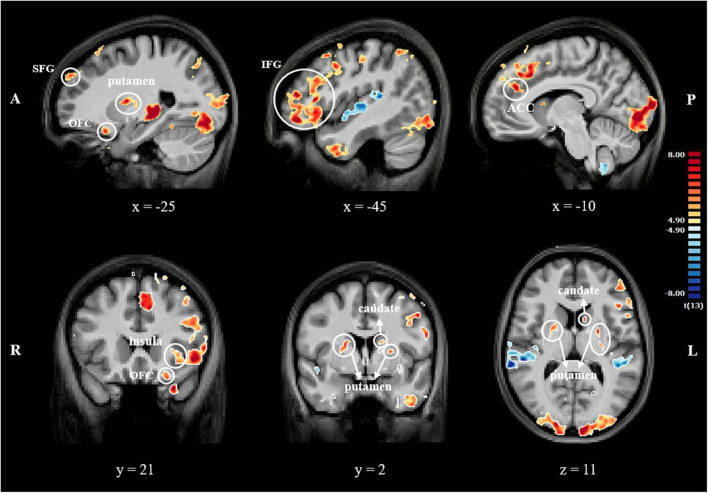
Series of sagittal, transversal and coronal slices showing BOLD response (FDR corrected, *q* = 0.01) for contrast 1. Coordinates reported in MNI space. The color bar represents *t*-values. A, anterior; P, posterior; R, right; L, left; SFG, superior frontal gyrus; IFG, inferior frontal gyrus; OFC, orbital frontal cortex; ACC, anterior cingulate cortex.

Regarding our hypothesized areas, significant activation clusters were found in the left superior frontal gyrus (SFG), IFG, and left OFC. We found bilaterally clusters with significant activation in the putamen and left caudate nucleus. Significant activation was also found in the left anterior insula and left supracallosal ACC (Table 2 and Figure 2).

**TABLE 2:** Brain regions activated in contrast self-criticism + avoidance + rumination + self-reassurance > baseline, and beta weights for each condition in each cluster.

Region	No.	Coordinates (MNI)			Cluster size voxel	Self-criticism		Avoidance		Rumination		Self-reassurance	
		X	Y	Z		Beta	*t*	Beta	*t*	Beta	*t*	Beta	*t*
L Superior frontal gyrus, dorsolateral	3	−14	51	43	649	0.47	6.79	0.37	9.8	0.38	6.25	0.33	9.6
		−23	53	35	1335	0.41	7.58	0.36	8.02	0.37	7.68	0.34	8.25
		−21	27	58	440	0.32	6.24	0.34	8.4	0.33	5.3	0.36	7.33
L Inferior frontal gyrus, triangular part	9	−46	44	12	792	0.48	7.6	0.43	7.7	0.44	7.5	0.4	6.58
		−50	22	−2	2551	0.58	8.95	0.55	7.94	0.53	10.04	0.55	9.35
		−45	42	−3	2580	0.44	9.32	0.39	9	0.37	8.59	0.35	11.18
		−47	23	25	2359	0.42	7.57	0.38	9.42	0.39	8.06	0.36	8.99
		−50	26	9	723	0.27	8.01	0.26	6.13	0.24	6.77	0.23	7.33
L Inferior frontal gyrus, pars orbitalis	11	−45	43	−10	1622	0.42	8.81	0.37	9.37	0.37	8.22	0.35	9.34
L Posterior orbital gyrus	29	−25	21	−19	417	0.31	6.14	0.3	8.4	0.32	6.03	0.3	4.4
L Insula	33	−35	21	−4	601	0.23	6.33	0.19	7	0.21	7.8	0.19	5.43
L Caudate nucleus	75	−11	10	11	101	0.17	4.81	0.19	4.67	0.17	5.46	0.16	3.93
		−16	2	17	118	0.2	4.29	0.23	5.31	0.22	5.18	0.19	4.5
L Lenticular nucleus, Putamen	77	−24	−3	8	835	0.17	6.31	0.18	8.03	0.16	6.65	0.17	6.79
R Lenticular nucleus, Putamen	78	20	0	13	669	0.14	4.7	0.15	6.55	0.15	7.7	0.12	5.09
L Anterior cingulate cortex, supracallosal	155	−9	37	27	581	0.23	7.51	0.22	7.01	0.24	7.74	0.18	4.58

Multiple clusters with the same label are shown in subsequent lines. Regions are labeled according to the AAL3 atlas. No. = Label number for the anatomical region according to the AAL3 atlas. Centre of gravity coordinates are reported. R, right; L, left.

#### 3.3.2 Contrast 2: self-criticism + avoidance + rumination + self-reassurance > 1-back task

**FIGURE 3:**
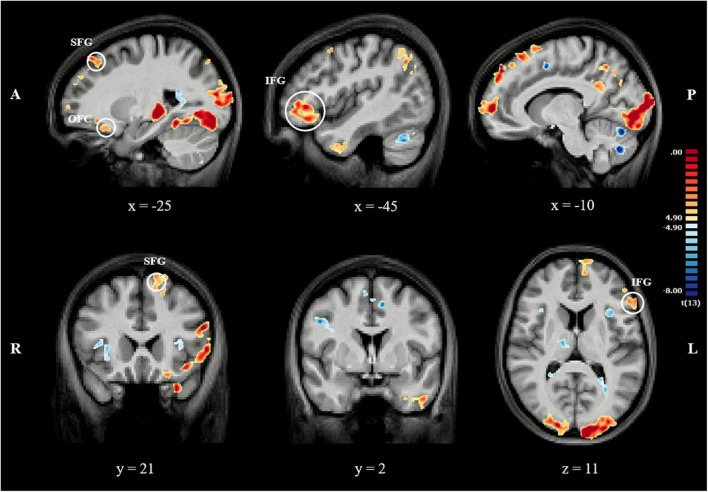
Series of sagittal, transversal and coronal slices showing BOLD response (FDR corrected, q = 0.01) for contrast 2. Coordinates reported in MNI space. The color bar represents *t*-values. A, anterior; P, posterior; R, right; L, left; SFG, superior frontal gyrus; IFG, inferior frontal gyrus; OFC, orbital frontal cortex.

As can be seen in Figure 3, when contrasting the four conditions against the 1-back task, specifically for the areas of interest in this study, we observed stronger activation in SFG, IFG and OFC. Conversely, this contrast revealed overlapping activation in the insula, striatum and ACC. Overall, contrast 2 revealed similar results to contrast 1.

#### 3.3.3 Comparison between self-criticism, avoidance, rumination, and self-reassurance

When contrasting the four different conditions between them, no difference was found. Also, the results of the two-way repeated measures ANOVA revealed that there was a significant main effect of ROIs, *F*_(7,91)_ = 16.59; *p* < 0.001; η^2^*p* = 0.56, and a non-significant main effect of conditions on participants’ brain activity, *F*_(3,39)_ = 1.13; *p* = 0.348; η^2^*p* = 0.08. There was no significant interaction between ROIs and conditions, *F*_(21,273)_ = 1.15; *p* = 0.300; η^2^*p* = 0.08.

**FIGURE 4:**
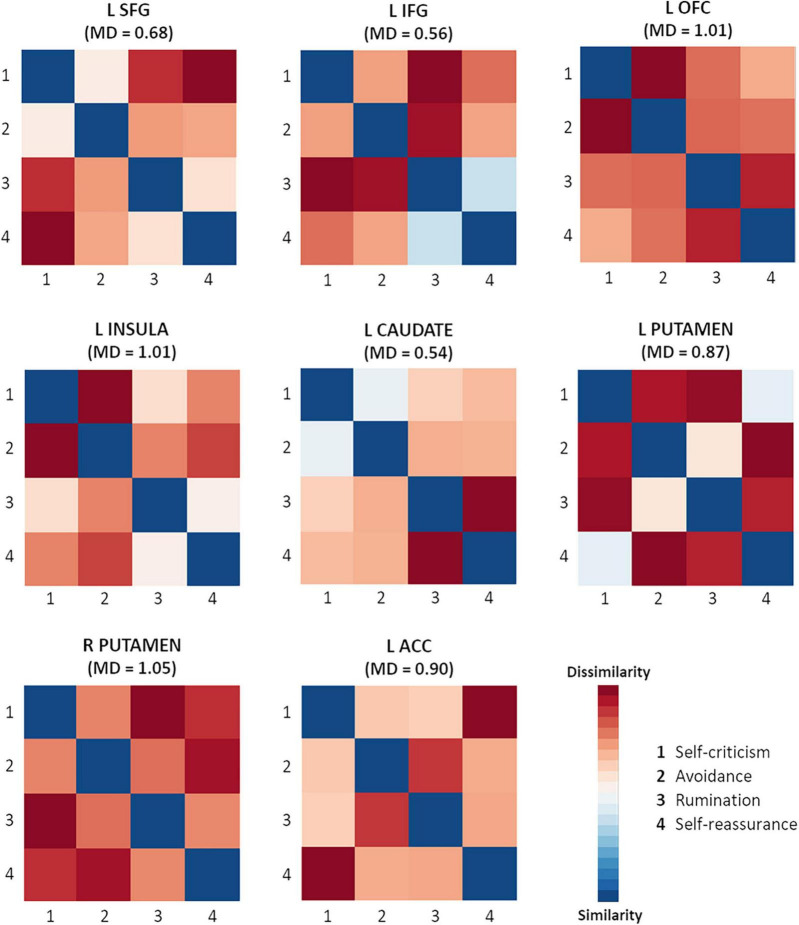
Dissimilarity matrix for the conditions for each area of interest. SFG, superior frontal gyrus; IFG, inferior frontal gyrus; OFC, orbital frontal cortex; ACC, anterior cingulate cortex; L, left; R, right; MD, maximum of dissimilarity (the highest value of the dissimilarity matrix).

To understand if condition related differences could be captured using representational similarity analysis (RSA), we computed the respective plots for each ROI, which are depicted in Figure 4. In general, there was a surprisingly low degree of similarity across conditions in all regions, a pattern that was not captured by standard GLM analysis. The dissimilarity was statistically significant from 0, for each pair of conditions for all ROIs (Table 3, showing statistical inference with corrections for multiple comparisons). This shows that in spite of the fact that self-relevant processing recruits the same regions across conditions, their pattern is fundamentally different.

**TABLE 3:** Bonferroni’s adjusted *p*-values of dissimilarity (against zero) for pairs of conditions for each ROI.

	Self-criticism × avoidance	Self-criticism × rumination	Self-criticism × self-reassurance	Avoidance × rumination	Avoidance × self-reassurance	Rumination × self-reassurance
L SFG	<0.001	0.003	<0.001	<0.001	<0.001	<0.001
L IFG	0.009	0.001	0.007	<0.001	<0.001	<0.001
L OFC	<0.001	<0.001	<0.001	<0.001	<0.001	<0.001
L Insula	0.005	<0.001	<0.001	<0.001	<0.001	<0.001
L Caudate	0.006	0.004	<0.001	0.002	0.005	0.005
L Putamen	<0.001	<0.001	<0.001	<0.001	<0.001	<0.001
R Putamen	<0.001	<0.001	<0.001	<0.001	<0.001	<0.001
L ACC	0.011	0.002	<0.001	<0.001	0.003	<0.001

SFG, superior frontal gyrus; IFG, inferior frontal gyrus; OFC, orbitofrontal gyrus; ACC, anterior cingulate cortex; L, left; R, right.

### 3.4 Correlations between the signal of regions of interest and self-reported psychological measures

Table 4 shows the exploratory correlational analyses between the signal of areas of interest and self-reported measures. We found positive correlations between inadequate self scores and activation in the left SFG (self-criticism and avoidance conditions), as well as in the left insula for the rumination condition. Food thought suppression scores were positively associated with activation in the left putamen during the self-reassurance condition, and rumination scores were positively correlated with activation in the SFG (rumination condition). Furthermore, self-compassion scores correlated negatively with the activation in the left SFG (in the avoidance condition), left insula in the ruminative and self-reassurance conditions, and right putamen for the self-reassurance condition. Reassured self scores were negatively correlated with the posterior orbital gyrus (in the self-reassurance condition) and the left insula activation in the rumination condition. Finally, scores in eating psychopathology were positively correlated with activations in the left SFG for the avoidance condition, left insula (avoidance and rumination conditions), left putamen (rumination and self-reassurance conditions) and right putamen for self-reassurance condition.

**TABLE 4:** Correlations between brain regions and self-reported psychological measures.

	FSCRS Inadequate	FTSI	RRS-ED	SCS	FSCRS Reassured	EDE-Q
**L Superior frontal gyrus, dorsolateral**						
Self-criticism	0.62*	0.21	0.03	−0.32	−0.30	0.35
Avoidance	0.60*	0.45	0.47	−0.53*	−0.20	0.59*
Rumination	0.17	0.39	0.62*	−0.39	−0.14	0.19
Self-reassurance	0.32	0.44	0.29	−0.26	0.03	0.38
**L Inferior frontal gyrus, pars orbitalis**						
Self-criticism	−0.06	−0.10	−0.21	0.09	0.08	−0.06
Avoidance	−0.21	0.30	−0.11	0.21	0.50	−0.15
Rumination	0.20	0.24	0.15	−0.14	−0.25	0.14
Self-reassurance	−0.15	0.13	0.06	−0.09	0.03	0.12
**L Posterior orbital gyrus**						
Self-criticism	0.29	0.20	0.21	−0.44	−0.46	0.40
Avoidance	0.08	0.15	0.11	−0.19	−0.07	0.53
Rumination	0.14	−0.05	0.08	−0.04	−0.23	0.40
Self-reassurance	0.37	0.13	0.29	−0.49	−0.54*	0.32
**L Insula**						
Self-criticism	0.22	0.12	0.20	−0.30	−0.15	0.32
Avoidance	0.22	0.33	0.09	−0.31	0.17	0.56*
Rumination	0.59*	0.18	0.46	−0.70**	−0.68**	0.59*
Self-reassurance	0.29	0.19	0.45	−0.66*	−0.41	0.42
**L Caudate**						
Self-criticism	0.04	0.08	0.00	−0.13	−0.01	−0.07
Avoidance	0.08	0.07	0.03	−0.05	0.21	0.39
Rumination	0.29	0.21	0.27	−0.40	−0.40	0.18
Self-reassurance	0.16	0.32	0.16	−0.31	−0.27	0.32
**L Putamen**						
Self-criticism	0.34	0.37	0.12	−0.33	−0.06	0.33
Avoidance	0.26	0.32	−0.01	−0.15	0.23	0.39
Rumination	0.42	0.48	0.41	−0.40	−0.18	0.53*
Self-reassurance	0.41	0.60*	0.36	−0.41	−0.21	0.73**
**R Putamen**						
Self-criticism	0.22	0.05	0.08	−0.41	−0.12	0.21
Avoidance	0.14	0.01	−0.19	−0.14	0.14	0.29
Rumination	0.31	0.03	0.34	−0.37	−0.33	0.20
Self-reassurance	0.35	0.29	0.19	−0.63*	−0.41	0.58*
**L Anterior cingulate cortex, supracallosal**						
Self-criticism	0.14	−0.33	−0.03	−0.26	−0.27	−0.01
Avoidance	0.05	0.17	0.03	−0.11	0.10	0.44
Rumination	0.02	0.16	0.39	−0.29	−0.30	−0.13
Self-reassurance	−0.12	0.14	0.37	−0.05	−0.19	0.35

FSCRS, forms of self-criticizing/attacking and self-reassuring scale inadequate subscale; FTSI, food thought suppression inventory; RRS-ED, ruminative response scale for eating disorders; SCS, self-compassion scale; EDE-Q, eating disorder evaluation questionnaire; R, right; L, left.

*p < 0.05,

**p < 0.01.

## 4 Discussion

The present study aimed to investigate the neural underpinnings of cognitive strategies of self-criticism, avoidance, rumination and self-reassurance related to eating and body concerns using a novel fMRI task. Also, we further examined the associations between neural correlates of each condition with self-reported psychological measures.

The findings showed significant activations in the PFC (particularly SFG and IFG), insula and basal ganglia (putamen and caudate) for all conditions. These results are consistent with previous literature on the neural correlates of defensive strategies and self-reassurance/self-compassion (Longe et al., 2010; Brühl et al., 2014; Doerig et al., 2014; Carew et al., 2015). Contrast analysis between conditions did not reveal any significantly different activations, indicating that a similar neural network is activated at similar levels, as captured by GLM analysis, across all conditions. However, each condition was found to have a distinct pattern of processing in each area of interest, as shown by the RSA. While the standard GLM compares mean activations across conditions, RSA captures multivariate patterns of neural activity and compares whether those patterns are similar or dissimilar across conditions (Kriegeskorte, 2008). This reveals that self-criticism, rumination, avoidance and self-reassurance may be encoded differently over time in the same brain region, underlying distinct mental operations or cognitive demands.

Contrary to our expectation, there were no significant activations when contrasting self-criticism with self-reassurance, which is different from what previous studies have shown (Longe et al., 2010; Kim et al., 2020b). It is possible that the same neural regions process these strategies along a dimensional spectrum. Another possible reason for this divergence might be related to differences in experimental designs. While previous studies asked participants to imagine being self-critical or self-reassuring and what those thoughts would be, our task provided participants with predetermined content for these thoughts. Our aim with this design choice was to reduce variability in task performance and improve the overall validity of the experimental task.

Our task activated several key frontal regions, including dlPFC, ventrolateral PFC (VLPFC) and OFC, that have been implicated in inhibitory control (Aron et al., 2004; Phillips et al., 2008) and cognitive emotion regulation (Goldin et al., 2008; Ochsner et al., 2012). The dlPFC is responsible for downregulating the emotional response by inhibiting limbic regions and insula (Beauregard, 2007; Ochsner and Gross, 2008; Ochsner et al., 2012), while the vlPFC is responsible for emotional appraisal and connects directly with the dlPFC to regulate emotion via the anterior middle cingulate gyrus (Kohn et al., 2014). In line with Longe et al. (2010) findings, we found a positive correlation between inadequate self scores and dlPFC activation during both self-criticism and avoidance conditions, which suggests that highly self-critical individuals tend to engage more in error processing and inhibitory processes (Wittfoth et al., 2009; Nejati et al., 2021). This attempt to correct or solve an error and difficulties in inhibiting the repetitive cycle of thoughts could also explain the association found between self-reported rumination and the increased activation of the dlPFC in the rumination condition.

Activation of the OFC may reflect the important role of this region in emotion processing and subjective emotional experiences related to food and body stimuli. OFC and the orbitofrontal part of the IFG are both connected to the supracallosal ACC, areas related to punishment and reward, meaning that OFC computes the reward value and then sends the information to the ACC to modulate goal-related actions (Rolls et al., 2020a). The OFC activation in self-reassurance condition was negatively correlated with self-reassurance scores, indicating that difficulties in calming the self might require additional OFC recruitment in an attempt to modulate the affective state. This finding could have significant implications for therapeutic interventions, where promoting a self-reassured dialog may help in emotion processing (Gilbert and Procter, 2006).

We found activation in the anterior insula and ACC, which are known to be two hubs of the salience network. The anterior insula receives and integrates interoceptive-autonomic information to, along with ACC, guide individual responses and decisions (Seeley et al., 2007; Craig, 2009; Seeley, 2019). The co-activation of these regions occurs regardless of whether the salient stimuli are internal or external, and irrespective of their negative or positive valence (Bartra et al., 2013). Several studies have shown altered connectivity in the salience network in patients with psychiatric disorders like anorexia and bulimia nervosa, binge eating, schizophrenia, anxiety and depression (White et al., 2010; Pannekoek et al., 2013; Mcfadden et al., 2014; Sha et al., 2019; Stopyra et al., 2019). Therefore, it would be interesting to conduct the present task in patients with eating disorders or obesity to examine the activity in the salience network regions under cognitive processes related to eating, weight and body image. Interestingly, Longe et al. (2010) reported activation in insula only for the self-reassurance condition, but not for self-criticism. However, Doerig et al. (2014), which used individually tailored unpleasant stimuli, found insula activation in self-criticism. In our study, the anterior insula is found to be recruited in all four conditions, which suggests the salience of food and body-related stimuli to the self, thus greater emotional monitoring is involved. Nonetheless, the correlational analyses revealed a significant negative correlation between insula activation in the self-reassurance condition and self-compassion scores. Self-compassion involves being sensitive to our own suffering and having a commitment to alleviating it (Gilbert, 2014), and the role of the insula in the perception of the body’s internal state may contribute to the embodied nature of compassion.

The results also show a significant activation in the basal ganglia, specifically bilateral putamen and the left caudate nucleus. The dorsal striatum activation is known for its activation in studies related to the processing of reward and punishment processing (Pagnoni et al., 2002; Delgado et al., 2003). As also suggested by Longe et al. (2010), the defensive strategies elicited in this study may be associated with a form of self-punishment, as strategies focused on food and body concerns can induce feelings of inferiority and self-deprecation. However, we also found that these structures were activated in the self-reassurance condition, suggesting that negative emotional states due to defensive strategies may at least overlap the positive reward associated with a self-reassured response. Neurobehavioral correlational analyses revealed that individuals who scored higher on food thought suppression exhibited higher activation in the left putamen in the self-reassurance condition. This finding may indicate that when one repeatedly attempts to control their thoughts about food to control food intake, the elicited self-reassurance response (a new habitual response to be acquired) may compete with cognitive control processes, and thus enhance putamen activation (Robertson et al., 2015). Conversely, when individuals higher in self-compassion engage in a self-reassured dialog, they may not appraise the situation as interfering and the task may not require as much mental effort and emotional regulation, diminishing putamen activation (Otto et al., 2014; Robertson et al., 2015; André et al., 2019).

We observed a distinct pattern of lateralization during the execution of the task. The activated regions were predominantly located in the left hemisphere, which is strongly associated with language processing and inner speech production (Knecht, 2000; Morin and Hamper, 2012). As we asked participants to engage in internal dialog, this pattern of activation suggests that language processing plays a crucial role in the cognitive mechanisms engaged in our experimental task.

In contrast to the 1-back task, the four cognitive conditions revealed stronger activation in the SFG, IFG, and OFC, while the remaining brain areas of interest were equally involved in both cognitive strategies and the working memory task. The stronger activation of the PFC during the cognitive strategies is consistent with previous research that suggests the involvement of higher-level cortical structures in emotion regulation (Dixon et al., 2017; Berboth and Morawetz, 2021). This finding suggests that specific brain regions, particularly areas of the PFC, have a distinct role in cognitive strategies, which supports the use of this paradigm to assess eating and body-related cognitive strategies.

The present study may be limited by its relatively low sample size and it is essential to interpret the reported findings in consideration of this factor, and in particular that replication is needed. This is mitigated by the fact that fMRI studies have effect sizes which still allowed us in this case to employ random effects analysis. Moreover, we implemented correction for multiple comparisons. Nevertheless, we acknowledge that for generalization future studies with larger sample sizes will be needed. Also, our results are confined to a population of healthy individuals. Furthermore, the task involves participants evaluating their emotional states, implicating the monitoring of their internal states, which could influence neural activity. The correlation analysis was mainly exploratory, and while it provides valuable insights, further research is warranted to replicate the results observed in this study. The assessment of participants’ engagement in the cognitive processes is an inherent limitation which was mitigated by online experiential debriefing, where participants rated their performance in the engagement task.

Considering the tendency to use defensive cognitive strategies to cope with difficult emotions (Aldao et al., 2010; Prefit et al., 2019; Rodrigues et al., 2022), it would be of particular interest in future studies to test this paradigm with a sample of individuals with eating difficulties. Such study could lead to a deeper understanding of these self-relating cognitive processes and highlight potential differences between self-reassurance and defensive strategies. Moreover, it could provide better insight into the associations between eating psychopathology and brain activations found in our study. It would be interesting to examine the neural impact of a compassion-based intervention on individuals with eating disorders (Goss and Allan, 2010, 2014; Goss, 2011). Compassion approaches, designed to address self-criticism and shame, and promote the activation of the soothing and contentment system, could potentially alter the response patterns of the neural circuitry underlying those processes.

## 5 Conclusion

The present study is the first to examine the neural correlates of self-relevant cognitive processes such as self-criticism, avoidance, rumination and self-reassurance linked to food and body concerns. The four cognitive processes activated a similar set of brain regions such as superior and inferior frontal gyri, OFC, insula, dorsal striatum, and ACC, but with a differential pattern across conditions as shown by RSA. Neurobehavioral correlational analyses revealed quite distinct patterns across conditions, supporting the dissimilarity analysis. These findings contribute to a growing body of research on these regulation processes and encourage future studies to better understand how these processes differ between them.

## Data availability statement

The raw data supporting the conclusions of this article will be made available by the authors, without undue reservation.

## Ethics statement

The studies involving humans were approved by the Ethics Committee of the Faculty of Psychology and Educational Sciences of the University of Coimbra. The studies were conducted in accordance with the local legislation and institutional requirements. The participants provided their written informed consent to participate in this study.

## Author contributions

CM: Formal analysis, Conceptualization, Investigation, Visualization, Writing – original draft. AS: Methodology, Resources, Software, Validation, Visualization, Writing – review and editing. JC: Conceptualization, Investigation, Writing – review and editing. JD: Investigation, Methodology, Software, Writing – review and editing. PC: Conceptualization, Investigation, Supervision, Writing – review and editing. KG: Investigation, Supervision, Writing – review and editing. AP: Investigation, Supervision, Validation, Writing – review and editing. MC-B: Conceptualization, Data curation, Funding acquisition, Investigation, Project administration, Supervision, Validation, Visualization, Writing – review and editing.

## Footnotes

1
https://github.com/alexsayal/fmriprep2BV
