# Supplementary material for: A neural network underlying cognitive strategies related to eating, weight and body image concerns
Source: Front Hum Neurosci. 2024 Jan 22;17:1274817. doi: 10.3389/fnhum.2023.1274817 (PMC10839062; doi:10.3389/fnhum.2023.1274817)
Supplement: Supplementary file 1 [file Table_1.DOCX]

**Supplementary material for the article:**

**A neural network underlying cognitive strategies related to eating, weight and body image concerns**

***fMRIPrep***

***Anatomical data preprocessing***

A total of 1 T1-weighted (T1w) images were found within the input BIDS dataset. The T1-weighted (T1w) image was corrected for intensity non-uniformity (INU) with N4BiasFieldCorrection (Tustison et al., 2010), distributed with ANTs 2.3.3 (Avants et al., 2008, RRID:SCR_004757), and used as T1w-reference throughout the workflow. The T1w-reference was then skull-stripped with a *Nipype* implementation of the antsBrainExtraction.sh workflow (from ANTs), using OASIS30ANTs as target template. Brain tissue segmentation of cerebrospinal fluid (CSF), white-matter (WM) and gray-matter (GM) was performed on the brain-extracted T1w using fast (FSL 5.0.9, RRID:SCR_002823, Zhang et al., 2001). Volume-based spatial normalization to one standard space (MNI152NLin2009cAsym) was performed through nonlinear registration with antsRegistration (ANTs 2.3.3), using brain-extracted versions of both T1w reference and the T1w template. The following template was selected for spatial normalization: *ICBM 152 Nonlinear Asymmetrical template version 2009c* [(Fonov et al., 2009, RRID:SCR_008796; TemplateFlow ID: MNI152NLin2009cAsym)].

***Functional data preprocessing***

For each of the 5 BOLD runs found per subject (across all tasks and sessions), the following preprocessing was performed. First, a reference volume and its skull-stripped version were generated by aligning and averaging 1 single-band references (SBRefs). A B0-nonuniformity map (or *fieldmap*) was estimated based on two (or more) echo-planar imaging (EPI) references with opposing phase-encoding directions, with 3dQwarp Cox and Hyde (1997) (AFNI 20160207). Based on the estimated susceptibility distortion, a corrected EPI (echo-planar imaging) reference was calculated for a more accurate co-registration with the anatomical reference. The BOLD reference was then co-registered to the T1w reference using flirt (FSL 5.0.9, Jenkinson and Smith, 2001) with the boundary-based registration (Greve and Fischl, 2009) cost-function. Co-registration was configured with nine degrees of freedom to account for distortions remaining in the BOLD reference. Head-motion parameters with respect to the BOLD reference (transformation matrices, and six corresponding rotation and translation parameters) are estimated before any spatiotemporal filtering using mcflirt (FSL 5.0.9, Jenkinson et al., 2002). BOLD runs were slice-time corrected using 3dTshift from AFNI 20160207 (Cox and Hyde, 1997, RRID:SCR_005927). First, a reference volume and its skull-stripped version were generated using a custom methodology of *fMRIPrep*. The BOLD time-series (including slice-timing correction when applied) were resampled onto their original, native space by applying a single, composite transform to correct for head-motion and susceptibility distortions. These resampled BOLD time-series will be referred to as *preprocessed BOLD in original space*, or just *preprocessed BOLD*. The BOLD time-series were resampled into standard space, generating a *preprocessed BOLD run in MNI152NLin2009cAsym space*. First, a reference volume and its skull-stripped version were generated using a custom methodology of *fMRIPrep*. Several confounding time-series were calculated based on the *preprocessed BOLD*: framewise displacement (FD), DVARS and three region-wise global signals. FD was computed using two formulations following Power (absolute sum of relative motions, Power et al., 2014) and Jenkinson (relative root mean square displacement between affines, Jenkinson et al., 2002). FD and DVARS are calculated for each functional run, both using their implementations in *Nipype* (following the definitions by Power et al., 2014). The three global signals are extracted within the CSF, the WM, and the whole-brain masks. Additionally, a set of physiological regressors were extracted to allow for component-based noise correction (*CompCor*, Behzadi et al., 2007). Principal components are estimated after high-pass filtering the *preprocessed BOLD* time-series (using a discrete cosine filter with 128s cut-off) for the two *CompCor* variants: temporal (tCompCor) and anatomical (aCompCor). tCompCor components are then calculated from the top 2% variable voxels within the brain mask. For aCompCor, three probabilistic masks (CSF, WM and combined CSF+WM) are generated in anatomical space. The implementation differs from that of Behzadi et al. in that instead of eroding the masks by 2 pixels on BOLD space, the aCompCor masks are subtracted a mask of pixels that likely contain a volume fraction of GM. This mask is obtained by thresholding the corresponding partial volume map at 0.05, and it ensures components are not extracted from voxels containing a minimal fraction of GM. Finally, these masks are resampled into BOLD space and binarized by thresholding at 0.99 (as in the original implementation). Components are also calculated separately within the WM and CSF masks. For each CompCor decomposition, the *k* components with the largest singular values are retained, such that the retained components’ time series are sufficient to explain 50 percent of variance across the nuisance mask (CSF, WM, combined, or temporal). The remaining components are dropped from consideration. The head-motion estimates calculated in the correction step were also placed within the corresponding confounds file. The confound time series derived from head motion estimates and global signals were expanded with the inclusion of temporal derivatives and quadratic terms for each (Satterthwaite et al., 2013). Frames that exceeded a threshold of 0.5 mm FD or 1.5 standardised DVARS were annotated as motion outliers. All resamplings can be performed with *a single interpolation step* by composing all the pertinent transformations (i.e. head-motion transform matrices, susceptibility distortion correction when available, and co-registrations to anatomical and output spaces). Gridded (volumetric) resamplings were performed using antsApplyTransforms (ANTs), configured with Lanczos interpolation to minimize the smoothing effects of other kernels (Lanczos, 1964). Non-gridded (surface) resamplings were performed using mri_vol2surf (FreeSurfer).

Many internal operations of *fMRIPrep* use *Nilearn* 0.6.2 (Abraham et al., 2014, RRID:SCR_001362), mostly within the functional processing workflow. For more details of the pipeline, see (<https://fmriprep.org/en/latest/workflows.html>).

**Supplementary Table 1**

Brain regions activated in contrast self-criticism + avoidance + rumination + self-reassurance > baseline, and beta weights for each condition in each cluster

| Region | No. | Coordinates (MNI) | | | Cluster size voxel | Self-criticism | | Avoidance | | Rumination | | Self-reassurance | |
| --- | --- | --- | --- | --- | --- | --- | --- | --- | --- | --- | --- | --- | --- |
|  |  | X | Y | Z |  | Beta | *t* | Beta | *t* | Beta | *t* | Beta | *t* |
| L Precentral gyrus | 1 | -47 | 6 | 39 | 1229 | 0.41 | 6.66 | 0.43 | 6.81 | 0.39 | 8.59 | 0.38 | 8.16 |
| L Superior frontal gyrus, dorsolateral | 3 | -14 | 51 | 43 | 649 | 0.47 | 6.79 | 0.37 | 9.80 | 0.38 | 6.25 | 0.33 | 9.60 |
|  |  | -23 | 53 | 35 | 1335 | 0.41 | 7.58 | 0.36 | 8.02 | 0.37 | 7.68 | 0.34 | 8.25 |
|  |  | -21 | 27 | 58 | 440 | 0.32 | 6.24 | 0.34 | 8.40 | 0.33 | 5.30 | 0.36 | 7.33 |
| L Middle frontal gyrus | 5 | -40 | 15 | 52 | 911 | 0.31 | 9.76 | 0.27 | 8.48 | 0.27 | 8.61 | 0.23 | 6.85 |
| L Inferior frontal gyrus, triangular part | 9 | -46 | 44 | 12 | 792 | 0.48 | 7.60 | 0.43 | 7.70 | 0.44 | 7.50 | 0.40 | 6.58 |
|  |  | -50 | 22 | -2 | 2551 | 0.58 | 8.95 | 0.55 | 7.94 | 0.53 | 10.04 | 0.55 | 9.35 |
|  |  | -45 | 42 | -3 | 2580 | 0.44 | 9.32 | 0.39 | 9.00 | 0.37 | 8.59 | 0.35 | 11.18 |
|  |  | -47 | 23 | 25 | 2359 | 0.42 | 7.57 | 0.38 | 9.42 | 0.39 | 8.06 | 0.36 | 8.99 |
|  |  | -50 | 26 | 9 | 723 | 0.27 | 8.01 | 0.26 | 6.13 | 0.24 | 6.77 | 0.23 | 7.33 |
| L Inferior frontal gyrus, pars orbitalis | 11 | -45 | 43 | -10 | 1622 | 0.42 | 8.81 | 0.37 | 9.37 | 0.37 | 8.22 | 0.35 | 9.34 |
| L Supplementary motor area | 15 | -6 | 22 | 46 | 1761 | 0.34 | 9.03 | 0.35 | 7.08 | 0.34 | 8.71 | 0.30 | 8.62 |
| L Superior frontal gyrus, medial | 19 | -6 | 32 | 47 | 1879 | 0.29 | 9.63 | 0.29 | 6.68 | 0.26 | 7.02 | 0.23 | 6.99 |
|  |  | -5 | 51 | 38 | 706 | 0.34 | 8.03 | 0.27 | 7.74 | 0.29 | 6.08 | 0.24 | 6.46 |
| L Posterior orbital gyrus | 29 | -25 | 21 | -19 | 417 | 0.31 | 6.14 | 0.30 | 8.40 | 0.32 | 6.03 | 0.30 | 4.40 |
| L Insula | 33 | -35 | 21 | -4 | 601 | 0.23 | 6.33 | 0.19 | 7.00 | 0.21 | 7.80 | 0.19 | 5.43 |
| L Hippocampus | 41 | -27 | -28 | -2 | 3364 | 0.23 | 9.15 | 0.22 | 12.23 | 0.22 | 13.61 | 0.21 | 9.42 |
| R Hippocampus | 42 | 25 | -28 | -3 | 1702 | 0.22 | 9.02 | 0.23 | 11.12 | 0.24 | 9.63 | 0.23 | 7.90 |
| L&R Calcarine fissure and surrounding cortex | 47/48 | 0 | -87 | -7 | 5172 | 1.59 | 15.90 | 1.54 | 15.99 | 1.58 | 20.43 | 1.58 | 14.12 |
| R Cuneus | 50 | 16 | -99 | 6 | 1239 | 1.46 | 12.65 | 1.44 | 12.27 | 1.54 | 15.75 | 1.46 | 11.91 |
| L Middle occipital gyrus | 55 | -11 | -99 | 6 | 1609 | 1.79 | 14.00 | 1.73 | 16.44 | 1.79 | 17.05 | 1.71 | 16.17 |
|  |  | -30 | -88 | 16 | 989 | 0.57 | 8.42 | 0.56 | 8.68 | 0.60 | 10.72 | 0.57 | 7.94 |
| L Inferior occipital gyrus | 57 | -24 | -83 | -12 | 1879 | 1.16 | 13.76 | 1.08 | 12.68 | 1.15 | 13.69 | 1.11 | 11.73 |
| L Fusiform gyrus | 59 | -35 | -54 | -17 | 1484 | 0.38 | 7.75 | 0.36 | 8.90 | 0.37 | 9.59 | 0.38 | 8.37 |
| R Fusiform gyrus | 60 | 31 | -50 | -18 | 524 | 0.45 | 6.03 | 0.44 | 6.39 | 0.46 | 5.63 | 0.49 | 7.51 |
|  |  | 26 | -82 | -14 | 1689 | 1.28 | 13.32 | 1.19 | 12.63 | 1.25 | 14.19 | 1.23 | 10.66 |
| L Postcentral gyrus | 61 | -48 | -20 | 47 | 830 | 0.17 | 6.71 | 0.21 | 5.57 | 0.18 | 6.17 | 0.20 | 13.20 |
| L Superior parietal gyrus | 63 | -24 | -73 | 44 | 437 | 0.32 | 4.64 | 0.35 | 6.44 | 0.35 | 5.91 | 0.32 | 6.51 |
| L Inferior parietal gyrus (excluding supramarginal and angular gyri) | 65 | -38 | -61 | 49 | 3075 | 0.34 | 7.86 | 0.32 | 6.90 | 0.32 | 9.64 | 0.26 | 6.57 |
| L Angular gyrus | 69 | -56 | -60 | 35 | 2107 | 0.38 | 7.97 | 0.33 | 6.28 | 0.34 | 9.53 | 0.28 | 7.38 |
| L Caudate nucleus | 75 | -11 | 10 | 11 | 101 | 0.17 | 4.81 | 0.19 | 4.67 | 0.17 | 5.46 | 0.16 | 3.93 |
|  |  | -16 | 2 | 17 | 118 | 0.20 | 4.29 | 0.23 | 5.31 | 0.22 | 5.18 | 0.19 | 4.50 |
| L Lenticular nucleus, Putamen | 77 | -24 | -3 | 8 | 835 | 0.17 | 6.31 | 0.18 | 8.03 | 0.16 | 6.65 | 0.17 | 6.79 |
| R Lenticular nucleus, Putamen | 78 | 20 | 0 | 13 | 669 | 0.14 | 4.70 | 0.15 | 6.55 | 0.15 | 7.70 | 0.12 | 5.09 |
| L Middle temporal gyrus | 89 | -60 | -42 | -10 | 763 | 0.25 | 6.52 | 0.22 | 7.48 | 0.23 | 8.76 | 0.20 | 10.86 |
| L Inferior temporal gyrus | 93 | -38 | 9 | -38 | 2899 | 0.34 | 8.59 | 0.29 | 11.34 | 0.32 | 9.66 | 0.27 | 11.64 |
| R Inferior temporal gyrus | 94 | 35 | 5 | -38 | 315 | 0.20 | 5.32 | 0.16 | 6.44 | 0.17 | 5.30 | 0.17 | 4.99 |
| L Crus I of cerebellar hemisphere | 95 | -36 | -73 | -27 | 640 | 0.38 | 8.94 | 0.30 | 6.25 | 0.33 | 9.13 | 0.29 | 6.79 |
| R Crus I of cerebellar hemisphere | 96 | 29 | -76 | -32 | 21539 | 0.48 | 12.95 | 0.42 | 12.00 | 0.41 | 21.28 | 0.42 | 10.02 |
| L Anterior cingulate cortex, supracallosal | 155 | -9 | 37 | 27 | 581 | 0.23 | 7.51 | 0.22 | 7.01 | 0.24 | 7.74 | 0.18 | 4.58 |

Multiple clusters with the same label are shown in subsequent lines. Regions are labeled according to the AAL3 atlas. No. = Label number for the anatomical region according to the AAL3 atlas. Center of gravity coordinates are reported. R = right; L = left
